# Corneal Cap Thickness and Its Effect on Visual Acuity and Corneal Biomechanics in Eyes Undergoing Small Incision Lenticule Extraction

**DOI:** 10.1155/2018/6040873

**Published:** 2018-06-28

**Authors:** Ting Liu, Ting Yu, Lina Liu, Kaijian Chen, Ji Bai

**Affiliations:** Department of Ophthalmology, Daping Hospital and Research Institute of Surgery, Third Military Medical University, Chongqing 400042, China

## Abstract

**Purpose:**

To evaluate the effect of corneal cap thickness on visual acuity and corneal biomechanics in small incision lenticule extraction (SMILE) for the treatment of myopia.

**Methods:**

Forty eyes of 20 patients undergoing SMILE for the treatment of myopia were enrolled in this prospective controlled study. The patients with 510 *μ*m–560 *μ*m central corneal thickness (CCT) and a refractive spherical equivalent of −3.00 D to −8.00 D were included. It was designed randomly to undergo SMILE with a 110 *μ*m cap thickness in one eye and 150 *μ*m cap thickness in the other. Ophthalmic examinations included best-corrected and uncorrected visual acuity (UCVA); refractive status, contrast sensitivity, and objective visual quality were evaluated at 2 h, 4 h, and 24 h postoperatively; while at 3 months after the procedure, corrected intraocular pressure (IOP), higher order aberrations (HOAs), and morphologic modifications of corneal architecture of both eyes were assessed.

**Results:**

Compared with the 150 *μ*m group, the incidence of OBL was significantly higher in the 110 *μ*m cap thickness group (*P*=0.004), and UCVA, Strehl ratio (SR), objective scatter index (OSI), modulation transfer function (MTF) cutoff frequency, and photopic and scotopic contrast sensitivity at medium spatial frequency were all significantly better in 110 *μ*m group at 2 h and 24 h postoperatively (*P* < 0.05). Corneal spherical aberration and corrected IOP by Corvis ST were significantly higher in the 110 *μ*m group at 3 months postoperatively (*P* < 0.05). No statistically significant differences were found in manifest refraction, UCVA, SR, OSI, MTF cutoff, and mesopic and photopic contrast sensitivity at low frequency, photopic contrast sensitivity at high frequency, endothelial density, corneal coma, and total HOAs at 3 months after the procedure. No visual decline was found in the patients in this study.

**Conclusions:**

Both 110 *μ*m and 150 *μ*m cap thickness in SMILE were safe and effective for treatment of myopia. A 110 *μ*m cap thickness demonstrated better visual outcomes during early and late postoperative periods with higher corneal spherical aberration and corrected IOP at 3 months postoperatively. This trial is registered with ChiCTR-IOR-17013369.

## 1. Introduction

Small incision lenticule extraction (SMILE) is a flapless, all-femtosecond laser refractive procedure, whereby the refractive lenticule is removed through a 2-3 mm keyhole incision. The safety, effectiveness, stability, and predictability of the procedure have made it a popular method for the treatment of myopia [1–4]. The SMILE procedure does not create a flap, thereby preserving more of the corneal nerve fibers, which has been shown to minimize dry eye and maintain higher corneal sensitivity [5]. As well, the postoperative corneal biomechanical strength is theoretically expected to be greater, when compared to LASIK and FS-LASIK [6]. In 2016, expert consensus from the Chinese Ophthalmology Association recommended that the standard cap thickness would be maintained between 110 and 120 *µ*m [1]. Theoretically, it would be expected that deeper cap thickness would maintain more anterior peripheral stroma and corneal nerve fibers with stronger corneal rigidity and faster recovery of ocular surface function. However, the effect of cap thickness on visual function outcomes and biomechanical characteristics of cornea after SMILE has not been fully assessed. Therefore, we did a randomized, prospective observation to assess the effect of two different cap thicknesses in SMILE for myopia treatment, especially in the first 24 h postoperative period.

## 2. Materials and Methods

Forty eyes of 20 patients undergoing SMILE for the treatment of myopia were enrolled in this prospective controlled study between August and November 2016 in Daping Hospital, Research Institute of Surgery, Third Military Medical University. The study adhered to the tenets of the Declaration of Helsinki, consistent with Good Clinical Practices and local regulatory requirements. Written informed consent was obtained from all study subjects, and the protocols were reviewed and approved by the institutional review board of Daping Hospital, Research Institute of Surgery, Third Military Medical University.

We recruited patients with stable myopic refractive error, aged 18 years or older, and the central corneal thickness (CCT), bilaterally, between 510 *μ*m and 560 *μ*m, and the manifest spherical equivalent between −3.00 D and −8.00 D with <1.50 D cylinder.

Patients with complicated systemic diseases such as diabetes, hypertension, heart disease, or connective tissue disorder or ocular diagnoses including amblyopia, anisometropia, keratoconus, central corneal residual stromal bed thickness <280 *μ*m, or history of other ocular pathology were excluded from the study.

Patients were required to suspend soft contact lens wear prior to their SMILE procedure. Basic soft contact lenses were forbidden for a week or more, more than 3 weeks for astigmatic correcting contacts lenses, and more than 3 months for orthokeratology lenses.

The randomisation was done via a coin toss method. One eye of each patient was randomized to SMILE procedure with a 110 *μ*m cap thickness and the other eye with a 150 *μ*m cap thickness. Each patient represented their own binocular control in order to minimize clinical, lifestyle, or environmental confounding factors.

All patients received routine preoperative topical antibiotic eye drops (0.3% tobramycin eye drops), twice daily for three days. Preoperative surface anesthesia was administered, (0.4% oxybuprocaine hydrochloride eye drops [benoxil; Santen Pharmaceuticals, Japan]) prior to the start of the procedure. Surgery in both eyes of the patients was performed by a single, experienced surgeon using the VisuMax femtosecond laser system (Zeiss, Germany) at power setting of 500 kHz, and cumulative energy 150–160 nJ. The thickness of the cap was intended to be 110 *μ*m in one eye and 150 *μ*m in the other eye, and the rest of the surgical parameters remained the same in the two groups. The refractive lenticule's diameter of the two groups was 6.3–6.5 mm, the thickness of the basal cornea was 10 *μ*m, and the side cut for accessing the lenticule was 90° at a circumferential width of 2 mm.

Most of the patients included in this study were less than 30 years of age; therefore, in order to prevent postoperative physiological regression of refractive status, the nomogram value of the spherical lens was increased by −0.50 D in the 110 *μ*m cap thickness group and −0.75 D in the 150 *μ*m thickness group (but the original value of the spherical lens which does not include the nomogram value was used in the statistical analysis). We usually calculate lenticule thickness extracted by sphere, namely correction of −1.00 D would consume 12-13 *μ*m of corneal stroma). The nomogram value of cylindrical lens remained unchanged. Postoperatively, conventional anti-inflammatory treatment was administered (0.5% loteprednol etabonate ophthalmic suspension, Bausch & Lomb, USA; four times daily for one week, followed by twice daily for 3-4 weeks).

Study assessments included preoperative and postoperative (2 hours, 4 hours, 1 day, and 3 months) slit lamp examinations, evaluation of uncorrected visual acuity (UCVA), refractive status, contrast sensitivity, and objective visual quality (SR, OSI, and MTF cutoff). And at three months postoperatively, some additional assessments were used, including corrected intraocular pressure (IOP) by Corvis ST (Oculus, Germany), corneal higher order aberrations (HOAs) by Pentacam HR (Oculus, Germany), anterior segment optical coherence tomography (AS-OCT, Carl Zeiss, Germany), confocal laser microscopy (HRT3, Heidelberg, Germany), and endothelial microscope (Tomey EM3000, Japan).

Statistical analysis was performed using Stata 13.1 (StataCorp, United States of America). Data with a normal distribution were represented by mean ± standard deviation (SD), otherwise by Q2 ± IQR; statistical difference of parameters between the two groups was calculated using a paired *t*-test or Fisher's exact test. *P* < 0.05 was considered statistically significant.

## 3. Results

Forty eyes of 20 patients (12 male and 8 female) were included in this study. The average age was 22.9 years. Preoperative and postoperative parameters at 3 months are given in Table 1. There were no significant differences in terms of preoperative parameters between the 2 groups, except for the preoperative refraction (*P* < 0.001). The 110 *μ*m group showed a sphere measurement of approximately 0.50 D higher than the 150 *μ*m group, which might be bias of the study.

All patients completed assessments up to the 3-month follow-up. During the follow-up, no severe complications were observed such as epithelial cell implantation inside the incision, cap rugs, diffuse lamellar keratitis (DLK), severe dry eye, transient photosensitivity syndrome, or ectasia. However, moderate or greater opaque bubble layer (OBL) was observed in 7 of 20 eyes (35%) in the 110 *μ*m group, while none was observed in the 150 *μ*m group (*P*=0.004).

**Figure 1:**
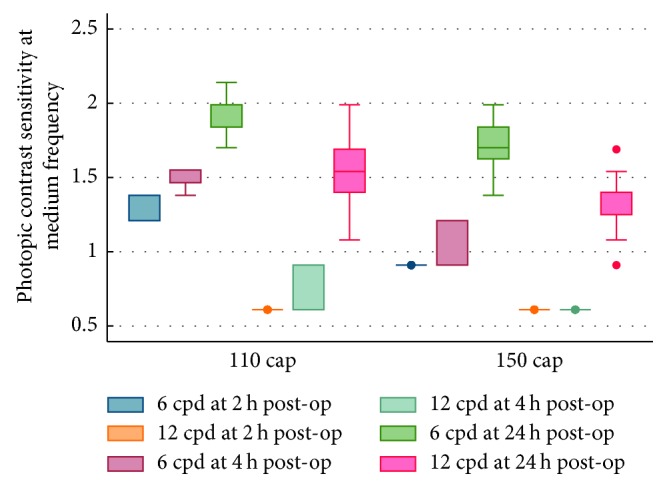
Photopic contrast sensitivity at medium frequency at 2, 4, and 24 hours postoperatively between the groups.

**Figure 2:**
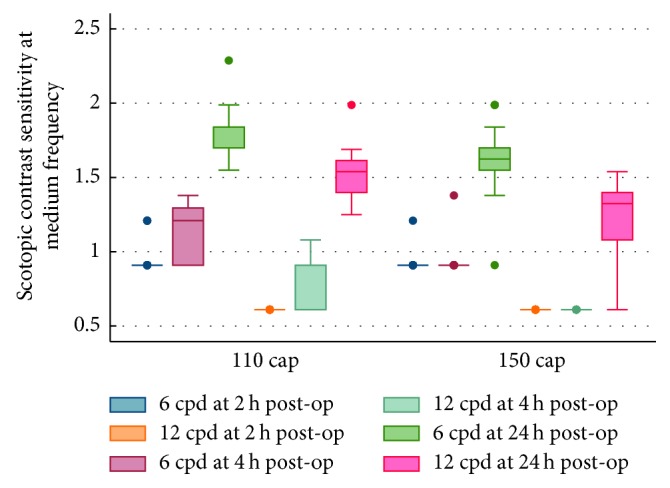
Scotopic contrast sensitivity at medium frequency at 2, 4, and 24 hours postoperatively between the groups.

UCVA, Strehl ratio (SR), objective scatter index (OSI), and modulation transfer function (MTF) cutoff frequency results were better in the 110 *μ*m group than in the 150 *μ*m group at 2, 4, and 24 hours postoperatively (all *P* < 0.05) (Table 2), although no statistically significant difference was found in OSI at 4 hours between the two groups (*P*=0.06). As shown in Table 2, optical quality analysis system II (OQAS, Visiometrics, Terrassa, Spain) related objective visual quality indicators (SR, OSI, and MTF cutoff) increased with time. Figures 1 and 2 revealed the changes in contrast sensitivity of medium spatial frequency (6 cpd and 12 cpd) under photopic and scotopic vision conditions in the early postoperative period. The results showed that the contrast sensitivity of the 110 *μ*m group was better than that of the 150 *μ*m group at medium spatial frequency under photopic or scotopic vision conditions.

At three months postoperative, as shown in Table 1, there was no marked difference in UCVA, spherical and cylindrical lens, SR, OSI, MTF cutoff, low-frequency photopic vision contrast sensitivity, corneal coma and HOAs, or endothelial cell density between the two groups (all *P* > 0.05). However, the 110 *μ*m group showed better results in high- and low-frequency scotopic contrast sensitivities, high-frequency photopic contrast sensitivities (*P* < 0.05), and larger corneal spherical aberration (*P*=0.003). IOP was measured at the 3-month postoperative visit using Corvis ST; the corrected IOP was higher in the 110 *μ*m group than the 150 *μ*m group (*P* < 0.001), whereas no statistically significant difference was found in any of the other corneal biomechanical indexes (all *P* > 0.05) (Table 3).

**Figure 3:**
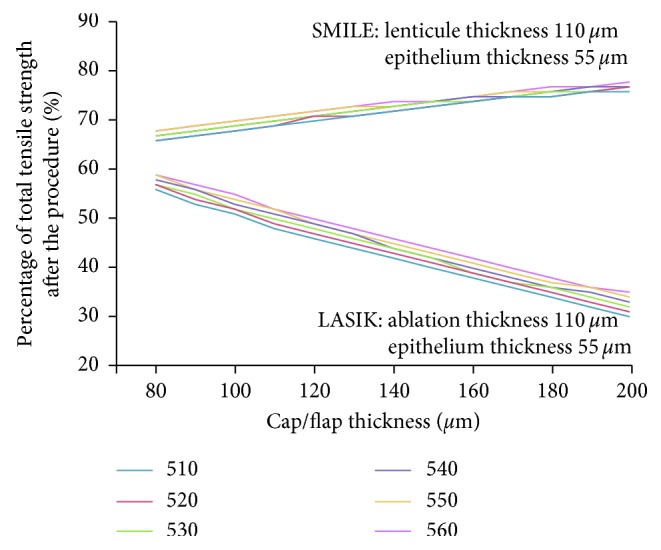
Change of total tensile strength after SMILE or LASIK with different CCT and cap/flap thickness (the different color of the lines represents different CCT with the same epithelium thickness of 55 *µ*m and the same cap/flap thickness of 110 *µ*m).

Figure 3 illustrates the relationship between the percentage of overall corneal tensile strength and different cap thickness/flap thickness after two common corneal refractive surgeries. Lines of different colors in the figure represent different CCT (510–560 *μ*m), but the epithelial thickness was set to 55 *μ*m, and the lens/cutting thicknesses of SMILE and LASIK were both set to 110 *μ*m. It can be seen that, under the premise of the same tissue thickness reduction, the overall postoperative corneal strengths of SMILE and LASIK were both reduced (<100%) compared with those before the surgery.

**Figure 4:**
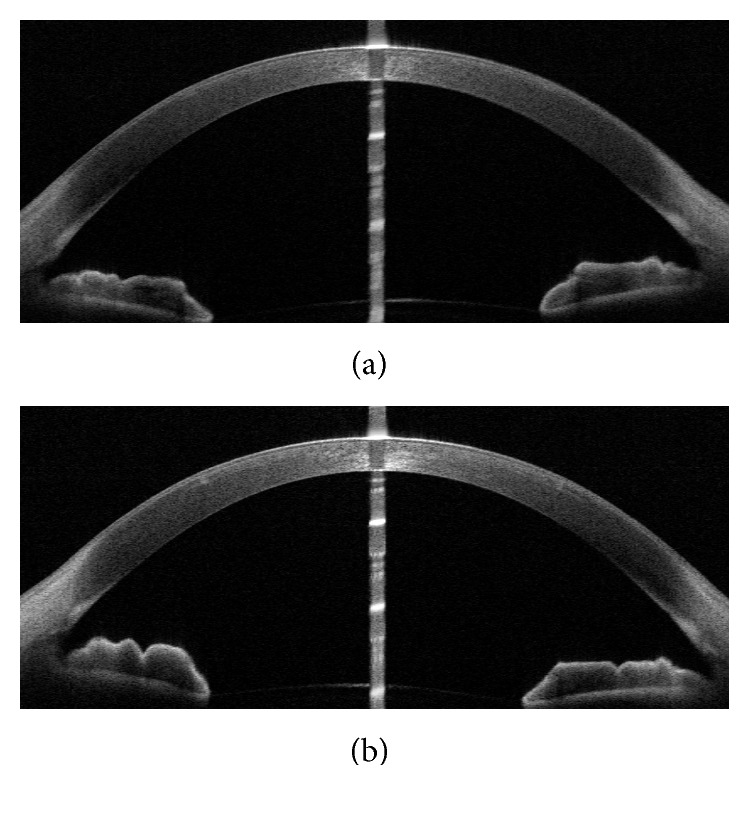
Three-month-postoperative examination of AS-OCT for one patient who underwent SMILE (yellow arrow represents interstromal space after the removal of lenticule and white arrow head shows the microfold of Bowman's layer). (a) OD (cap thickness 110 *μ*m), (b) OS (cap thickness 150 *μ*m).

Anterior segment optical coherence tomography (AS-OCT) examination provided images of the cornea of the eyes at 3 months postoperatively (Figure 4). Hyperreflective lines of interlaminar space could be clearly seen in a patient for both eyes, and the microfold of Bowman's membrane was particularly visible in his right eye with 110 *μ*m cap thickness (Figure 4).

**Figure 5:**
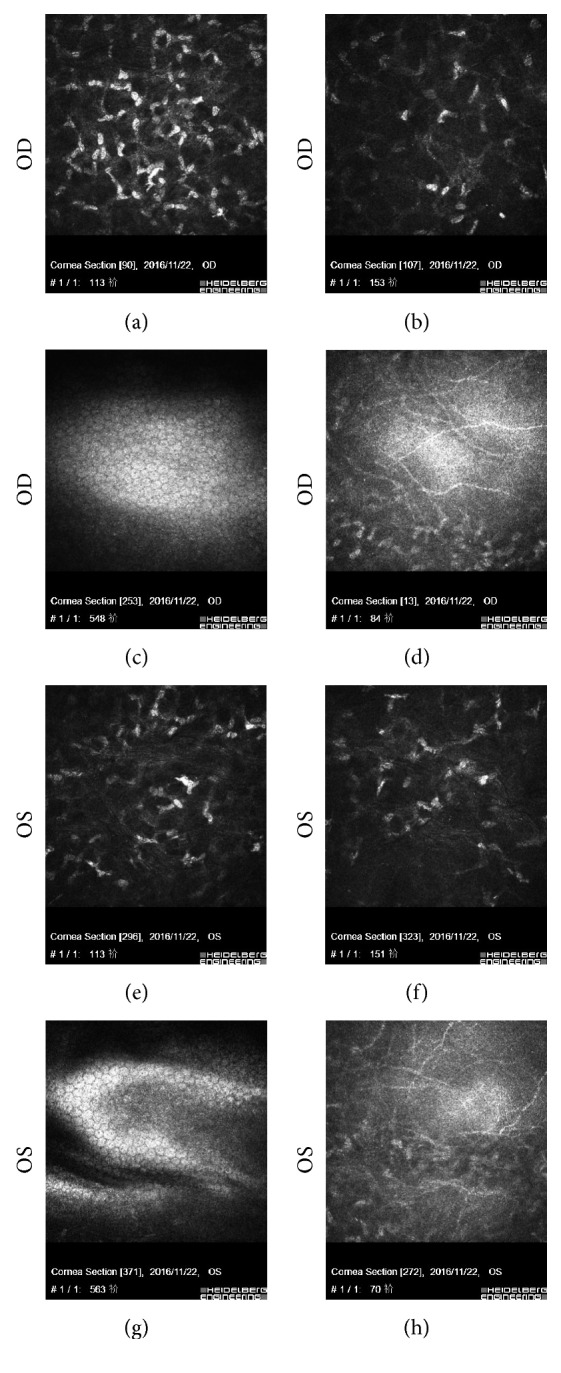
Three-month-postoperative examination of confocal laser corneal microscopy for the same patient who underwent SMILE (more active keratocytes around the depth of 110 *μ*m in OD was shown, but active keratocytes around the depth of 150 *μ*m in OS was not so significant. Similar endothelium and nerve fiber distribution around the incision was also indicated). (a, e) 110 *μ*m, (b, f) 110 *μ*m, (c, g) 550 *μ*m, and (d, h) around the incision.

Confocal microscope was used to evaluate the morphologic characteristics of the cornea. Some activated keratocytes were observed at sites of 2 types of cap, but the very bright keratocytes were more frequently visible at the depth of 110 *μ*m in the eye with 110 *μ*m cap thickness (Figures 5(a) and 5(e)), while less at the depth of 150 *μ*m in the fellow eye (Figures 5(b) and 5(f)). The distribution of nerve fibers near the endothelial cells and incision was similar in both eyes (Figures 5(d) and 5(g)).

## 4. Discussion

At present, the ideal cap thickness for SMILE has no unified standard, and its effect on visual quality and corneal biomechanics has not been well studied. In this study, we performed SMILE on randomized eyes with a 110 *μ*m cap thickness and the other eye 150 *μ*m cap thickness to assess the influence of different cap thicknesses on visual function and corneal biomechanics at the very early stage (2 hours to 24 hours postoperatively) and the stable stage (3 months). Our study showed that patients with 110 *μ*m cap thickness had higher incidence of intraoperative moderate or greater OBL and faster recovery of visual acuity; however, eyes with 150 *μ*m cap thickness demonstrated more stable spherical aberration and lower corrected IOP.

In our study, the results showed that patients with 110 *μ*m cap thickness had higher incidence of intraoperative moderate or greater OBL than the 150 *μ*m cap thickness group. It did not appear to have any influence on visual quality in the early postoperative stage. Whereas, the 110 *μ*m group was markedly better than the 150 *μ*m group in UCVA, SR, OSI, MTF cutoff, and the contrast sensitivity of moderate spatial frequency in photopic and scotopic environments 2, 4, and 24 hours after the procedure. The occurrence of OBL in SMILE is likely associated with the properties of femtosecond laser that the CO_2_ and H_2_O produced by the laser-induced tissue rupture may accumulate in the corneal stroma. The compactness of corneal fibers makes it more difficult for the gas to overflow and consequently creating a diaphanous bubble layer in the corneal stroma. Usually, mild OBL does not impact the surgical process, whereas moderate and severe OBL, especially those in lateral episiotomy and rescanning after loss of suction, may increase the lens' dialyte resistance, thereby triggering problems such as prolonged recovery of postoperative vision and poor visual quality in patients. In our previous work, the “water infiltration” separating method in SMILE surgery was proposed to relieve the “negative injury” induced by lens separation and accelerate the postoperative recovery of visual quality of patients [7, 8].

In addition, SR, OSI, and MTF cutoff all improved with time for both groups at very early postoperative period. There were also significant differences between these two groups in terms of objective visual quality indexes after postoperative 2, 4, and 24 hours (all *P* < 0.05). Moreover, Figure 1 revealed changes in contrast sensitivity in moderate spatial frequency (6 cpd and 12 cpd, with an optimal comparative sensitivity) between the two groups in the early postoperative stage. This suggests that at the early postoperative time, the 110 *μ*m cap thickness group was better than the 150 *μ*m group in terms of contrast sensitivity at the two spatial frequencies. Similarly, changes under a scotopic environment also indicated that 110 *μ*m thickness had quicker postoperative recovery of visual quality (Figure 2). We proposed that the reason for the difference in visual quality maybe that relative ease of shape modification and draining of interlaminar liquid for the thinner cap thickness at very early stage postoperatively.

This study results demonstrated that there was no significant difference in terms of SE, corneal coma, corneal total HOAs, and endothelial cell density (all *P* > 0.05) between the two cap thickness groups. And, no visual decline was found in all the patients. No significant difference was noted in various objective visual quality indices of OQAS, although the 110 *μ*m group was still superior to the 150 *μ*m group in terms of contrast sensitivity with high- and low-frequency scotopic vision and high-frequency photopic vision (*P* < 0.05). This indicated that the 110 *μ*m group was superior to the 150 *μ*m cap thickness group in terms of refined resolving capacity, rough sensory capacity under scotopic vision, and the refined resolving capacity under photopic vision. Few studies have evaluated the differences in visual function following SMILE with different cap thicknesses. Liu et al. provides evidence that there was a lower level corneal wound-healing response after SMILE with a 140 *μ*m-cap than with a 120 *μ*m cap, although the cap thickness did not affect visual outcomes by 3 months postoperatively [9]. The study also showed no statistically significant difference in results of visual acuity, CS, total HOAs, or manifest refractive error in eyes with thick corneas between 120 *μ*m cap and 140 *μ*m cap at 3 months postoperatively [10].

The average postoperative stable corneal spherical aberration was 0.38 *µ*m in the 110 *μ*m group and 0.32 *µ*m in the 150 *μ*m group (*P*=0.003). This might be associated with higher concave-sphere degree (−4.77 versus −4.25, *P* < 0.001) and the relative flatting corneal surface remolding in the 110 *μ*m group. There was a microfold of Bowman's membrane of the right eye in the 110 *μ*m group three months after the procedure; however, the visual quality was not affected, which was consistent with the study conclusions of Yao and Luo et al. [11, 12].

Corvis ST was examined preoperatively and at three months postoperatively, we found that there was a statistically significant difference in postoperative-corrected IOP (Ehlers' formula) between the two groups (19.9 mmHg versus 19.0 mmHg; *P* < 0.001) among all corneal biomechanical indicators. The analysis indicated that it may be due to the fact that the thicker cap retained more of the anterior stroma with less effect on the corneal biomechanical properties, so it had little effect on the formula for correcting the IOP [13]. This theory has also been clarified by Reinstein et al. by using a mathematical model of the fourth-order polynomial [14], which was also shown in Figure 3 that with the increase of cap thickness, the corneal tensile strength showed an upward trend after SMILE. But with the increase of flap thickness, the tensile strength of corneal was significantly decreased after LASIK. However, He and colleagues compared the changes of biomechanical properties in rabbit eyes that underwent SMILE with 100 *μ*m and 160 *μ*m cap thicknesses [15]. They observed minimal differences in the corneal biomechanics between the two treatment groups. In this study, the corrected IOP was lower than that of 110 *μ*m group at 3 months postoperatively, which may support the hypothesis that the cap does not retain the same rigidity as an untouched stroma.

We acknowledge the limitations of this study, particularly the small size of our cohort (20 in each group) and the short follow-up period. We do feel the necessity of increase of the sample size to make more statistically definite conclusion in the future study. Moreover, we think in spite of short period of follow-up, the refractive state of the patients was stable at three months after SMILE procedure.

In summary, we found in this study that both 110 and 150 *μ*m corneal cap thickness groups were safe and effective for correction of myopia and astigmatism in the very early stage and stable period after SMILE, but the incidence of moderate or high OBL was higher in the 110 *μ*m group with faster recovery of visual quality in early postoperative stage; at 3 months postoperatively, the spherical aberration and corrected IOP were higher in the 150 *μ*m group. Additional simulation-based and clinical studies are needed to further clarify the effects of cap thickness of SMILE on patients' visual quality and corneal biomechanics.

## Figures and Tables

**Table 1:** Baseline characteristics of all eyes preoperatively and at 3 months postoperatively. (mean ± SD or Q2 ± IQR).

	110 *μ*m cap (*n*=20)	150 *μ*m cap (*n*=20)	*P* value
Pre-BCVA	0.1 ± 0.1	0.1 ± 0.1	0.16
Pre-sph (D)	−4.77 ± 1.26	−4.25 ± 1.31	**<0.001**
Pre-cyl (D)	−0.75 ± 0.43	−0.77 ± 0.48	0.88
Post-UCVA (LogMAR)	−0.10 ± 0.0	−0.10 ± 0.0	0.99
Post-sph (D)	0.31 ± 0.50	0.20 ± 0.61	0.41
Post-cyl (D)	0.11 ± 0.34	0.06 ± 0.45	0.73
Pre-CCT (µm)	549.8 ± 23.7	548.9 ± 24.7	0.48
Pre-K1 (D)	42.4 ± 1.1	42.4 ± 1.1	0.63
Pre-K2 (D)	43.5 ± 1.2	43.5 ± 1.2	0.74
OBL ≥ level 2 (eyes)^*∗*^	7	0	**0.004** ^a^
Pre-SR	0.24 ± 0.08	0.20 ± 0.07	0.18
Post-SR	0.19 ± 0.05	0.16 ± 0.04	0.08
Pre-OSI	0.68 ± 0.47	0.69 ± 0.42	0.09
Post-OSI	0.88 ± 0.54	0.99 ± 0.47	0.10
Pre-MTF cutoff (cpd)	36.85 ± 12.21	34.84 ± 12.08	0.64
Post-MTF cutoff (cpd)	33.82 ± 11.03	32.26 ± 8.63	0.49
Prephotopic contrast sensitivity at low frequency (cpd)^&^	1.62 ± 0.04	1.62 ± 0.04	0.99
Postphotopic contrast sensitivity at low frequency (cpd)	1.66 ± 0.03	1.62 ± 0.03	0.25
Prephotopic contrast sensitivity at high frequency (cpd)^@^	1.17 ± 0.04	1.11 ± 0.03	0.28
Postphotopic contrast sensitivity at high frequency (cpd)	1.24 ± 0.04	1.08 ± 0.04	**0.001**
Prescotopic contrast sensitivity at low frequency (cpd)	1.62 ± 0.04	1.60 ± 0.02	0.57
Postscotopic contrast sensitivity at low frequency (cpd)	1.66 ± 0.05	1.51 ± 0.04	**0.004**
Prescotopic contrast sensitivity at high frequency (cpd)	1.05 ± 0.05	1.09 ± 0.05	0.38
Postscotopic contrast sensitivity at high frequency (cpd)	1.18 ± 0.04	1.05 ± 0.03	**0.009**
Precorneal spherical aberration (*µ*m)	0.17 ± 0.08	0.20 ± 0.08	0.10
Postcorneal spherical aberration (*µ*m)	0.38 ± 0.19	0.32 ± 0.10	**0.003**
Precorneal coma (*µ*m)^#^	0.20 ± 0.09	0.20 ± 0.07	0.36
Postcorneal coma (*µ*m)	0.41 ± 0.16	0.36 ± 0.20	0.32
Pre-higher-order aberration (*µ*m)	0.35 ± 0.08	0.36 ± 0.08	0.65
Post-higher-order aberration (*µ*m)	0.64 ± 0.14	0.62 ± 0.15	0.47
Preendothelia cell density (/mm^2^)	2343 ± 472	2369 ± 501	0.28
Postendothelia cell density (/mm^2^)	2321 ± 487	2336 ± 455	0.35

^*∗*^We classified intraoperative OBL into 3 levels, which are mild, moderate, and severe; low frequency, ^&^3 cpd; high frequency, ^@^18 cpd; BCDVA, best-corrected distance visual acuity; UCDVA, uncorrected distance visual acuity; SR, Strehl ratio; OSI, objective scatter index; MTF cutoff, modulate transfer function cutoff value; coma^#^ = [(C_7_) [2] + (C_8_) [2]]^1/2^. ^a^Fisher's exact test.

**Table 2:** Comparison of visual parameters in the early postoperative period. (2 h, 4 h, and 24 h) (mean ± SD).

Time point	Visual parameter	110 cap (*n*=20)	150 cap (*n*=20)	*P* value
2 h	UCVA	0.1 ± 0.0	0.50 ± 0.1	**<0.001**
	Sph (D)	0.78 ± 0.24	0.80 ± 0.42	0.88
	Cyc (D)	0.36 ± 0.16	0.30 ± 0.36	0.41
	SR	0.12 ± 0.04	0.08 ± 0.04	**0.01**
	OSI	2.71 ± 1.29	3.74 ± 1.43	**<0.001**
	MTF cutoff (cpd)	19.66 ± 5.61	15.55 ± 3.76	**0.01**

4 h	UCVA	0.1 ± 0.0	0.3 ± 0.1	**<0.001**
	Sph (D)	0.64 ± 0.24	0.63 ± 0.42	0.87
	Cyc (D)	0.14 ± 0.13	0.23 ± 0.17	0.05
	SR	0.14 ± 0.05	0.10 ± 0.03	**0.01**
	OSI	2.17 ± 0.96	2.65 ± 1.35	0.06
	MTF cutoff (cpd)	21.10 ± 6.12	17.15 ± 4.47	**0.01**

24 h	UCVA	0.1 ± 0.1	0.1 ± 0.1	**<0.001**
	Sph (D)	0.38 ± 0.30	0.36 ± 0.44	0.91
	Cyc (D)	0.20 ± 0.33	0.22 ± 0.55	0.89
	SR	0.21 ± 0.06	0.17 ± 0.08	**0.05**
	OSI	1.11 ± 0.95	1.96 ± 1.24	**0.01**
	MTF cutoff (cpd)	34.51 ± 11.31	24.76 ± 11.02	**0.01**

Uncorrected visual acuity (UCVA), Strehl ratio (SR), objective scatter index (OSI), and modulation transfer function (MTF) cutoff frequency.

**Table 3:** Comparison of intraocular pressure and other corneal biomechanical characteristics preoperatively and at month 3 (mean ± SD).

	110 cap (*n*=20)	150 cap (*n*=20)	*P* value
Pre-IOP (mmHg)	17.7 ± 1.4	17.9 ± 1.1	0.35
Post-IOP (mmHg) month 3	19.9 ± 1.5	19.0 ± 1.0	**<0.001**
Pre-AP1 (mm)	1.78 ± 0.05	1.73 ± 0.11	0.22
Post-AP1 (mm) month 3	1.61 ± 0.29	1.67 ± 0.20	0.59
Pre-AP2 (mm)	1.62 ± 0.37	1.67 ± 0.40	0.63
Post-AP2 (mm) month 3	1.34 ± 0.44	1.36 ± 0.44	0.83
Pre-PD (mm)	4.70 ± 0.92	3.83 ± 1.36	0.08
Post-PD (mm) month 3	3.98 ± 1.44	4.11 ± 1.46	0.82
Pre-DA (mm)	1.02 ± 0.05	1.00 ± 0.06	0.10
Post-DA (mm) month 3	1.09 ± 0.07	1.05 ± 0.24	0.59

AP1, the first applanation; AP2, the second applanation; PD, peak distance; DA, deformation amplitude.
